# Personalized ventilatory strategy based on lung recruitablity in COVID-19-associated acute respiratory distress syndrome: a prospective clinical study

**DOI:** 10.1186/s13054-023-04360-6

**Published:** 2023-04-19

**Authors:** Hiroki Taenaka, Takeshi Yoshida, Haruka Hashimoto, Andi Muhammad Fadlillah Firstiogusran, Suguru Ishigaki, Hirofumi Iwata, Yusuke Enokidani, Hironori Ebishima, Naoko Kubo, Moe Koide, Yukiko Koyama, Ryota Sakaguchi, Natsuko Tokuhira, Yu Horiguchi, Akinori Uchiyama, Yuji Fujino

**Affiliations:** The Department of Anesthesiology and Intensive Care Medicine, Osaka University Graduate School of Medicine, 2-15 Yamadaoka, Suita, Osaka 565-0871 Japan

**Keywords:** Acute respiratory distress syndrome, Lung recruitability, COVID-19, Electrical impedance tomography, Personalized ventilatory strategy

## Abstract

**Background:**

Heterogeneity is an inherent nature of ARDS. Recruitment-to-inflation ratio has been developed to identify the patients who has lung recruitablity. This technique might be useful to identify the patients that match specific interventions, such as higher positive end-expiratory pressure (PEEP) or prone position or both. We aimed to evaluate the physiological effects of PEEP and body position on lung mechanics and regional lung inflation in COVID-19-associated ARDS and to propose the optimal ventilatory strategy based on recruitment-to-inflation ratio.

**Methods:**

Patients with COVID-19-associated ARDS were consecutively enrolled. Lung recruitablity (recruitment-to-inflation ratio) and regional lung inflation (electrical impedance tomography [EIT]) were measured with a combination of body position (supine or prone) and PEEP (low 5 cmH_2_O or high 15 cmH_2_O). The utility of recruitment-to-inflation ratio to predict responses to PEEP were examined with EIT.

**Results:**

Forty-three patients were included. Recruitment-to-inflation ratio was 0.68 (IQR 0.52–0.84), separating high recruiter versus low recruiter. Oxygenation was the same between two groups. In high recruiter, a combination of high PEEP with prone position achieved the highest oxygenation and less dependent silent spaces in EIT (vs. low PEEP in both positions) without increasing non-dependent silent spaces in EIT. In low recruiter, low PEEP in prone position resulted in better oxygenation (vs. both PEEPs in supine position), less dependent silent spaces (vs. low PEEP in supine position) and less non-dependent silent spaces (vs. high PEEP in both positions). Recruitment-to-inflation ratio was positively correlated with the improvement in oxygenation and respiratory system compliance, the decrease in dependent silent spaces, and was inversely correlated with the increase in non-dependent silent spaces, when applying high PEEP.

**Conclusions:**

Recruitment-to-inflation ratio may be useful to personalize PEEP in COVID-19-associated ARDS. Higher PEEP in prone position and lower PEEP in prone position decreased the amount of dependent silent spaces (suggesting lung collapse) without increasing the amount of non-dependent silent spaces (suggesting overinflation) in high recruiter and in low recruiter, respectively.

**Supplementary Information:**

The online version contains supplementary material available at 10.1186/s13054-023-04360-6.

## Introduction

Since the first description of acute respiratory distress syndrome (ARDS) [1], many efforts have been made to identify ventilatory strategies minimizing ventilator-induced lung injury (VILI), but so far, majority of randomized clinical trials have failed to improve outcome [2]. This is probably because heterogeneous groups of patients from biological, physiological or morphological point of view are included to be a single clinical entity of ARDS [2, 3]. Recruitment-to-inflation (*R*/*I*) ratio has been recently developed as a simple bedside technique to identify the patients who has the potential for lung recruitment [4]. Thus, this technique may be potentially useful to identify patients with ARDS more likely to recruit in response to the specific intervention, i.e., higher PEEP, prone position, lung recruitment, and improve outcome.

Due to the coronavirus disease 2019 (COVID-19) pandemic, COVID-19-associated ARDS is rapidly expanding and its physiological features have been vigorously investigated [5–7]. It is reported that both respiratory system compliance and lung gas volume was higher in COVID-19-associated ARDS (vs. classical ARDS) when matching oxygenation [6]. Such discrepancy may be explained by a predominant contribution of intravascular pathology to hypoxemia in COVID-19-associated ARDS [8]. In some case series, PEEP settings on the basis of traditional oxygenation criteria, i.e., PEEP/F_I_O_2_ table were found to be misleading in COVID-19-associated ARDS, either insufficient to cause lung collapse or excessive to cause overinflation [9, 10]. These data suggest that apart from oxygenation criteria, ventilatory management for COVID-19-associated ARDS need to be tailored in each patient, depending on mechanical characteristics, in order to prevent VILI.

Therefore, we conducted a prospective study to evaluate if the impacts of PEEP (low and high) and body position (supine and prone) on lung mechanics and regional lung inflation (electrical impedance tomography [EIT]) may be different, depending on lung recruitability. Then we also examined the utility of *R*/*I* ratio to predict responses to PEEP with EIT. Part of data (two cases) has been previously published as a case report [11].

## Methods

Detailed methods are described in the Additional files 1, 2.

### Study population

This prospective observational study was carried out at Osaka University Hospital following the approval from the Ethics committee for Clinical Studies, Osaka University Hospital, Suita, Japan (No.20039). Forty-three consecutive patients were then enrolled in between May 2020 and February 2021. Patients were eligible if SARS-CoV-2 infection was positive, defined as being positive in real-time reverse transcriptase-polymerase chain reaction assay using nasal or pharyngeal swab samples; age was ${ \geqq }$ 18 years old; met criteria for ARDS *as per* the Berlin definition [12]. Patients reintubated after first enrollment were also included for additional measurements of EIT (Additional file 2: Figure S1).

### CT scans

Thoracic CT scans were obtained upon ICU admission in all patients. Unenhanced helical CT scans were performed. The volume of hyperinflation, normal aeration, poor aeration, and non-aeration was calculated, as previously described [13].

### Protocol

Prior to initiating the measurements, all patients were deeply sedated with sedatives and/or opioids and paralyzed with intravenous administration and a continuous infusion of rocuronium.

Patients were then sequentially assigned to each of four conditions as follows:

- High PEEP, Supine;
- Low PEEP, Supine;
- High PEEP, Prone;
- Low PEEP, Prone.

All patients were ventilated with assisted volume-controlled mode, targeting tidal volume of 6 ml/kg predicted body weight, respiratory frequency of 35 min^−1^ or less targeted to pH 7.20–7.45, inspiratory time of 0.6–1.2 s. Airway opening pressure was identified by a pressure–volume curve on the ventilator at low constant flow, as described previously [4]. High PEEP and low PEEP was defined as 15 cmH_2_O and 5 cmH_2_O (or airway opening pressure, either of which was higher) respectively. All measurements were conducted within 24 h after ICU admission and, for the patients who required reintubation, within 24 h after reintubation.

### Electric impedance tomography

EIT data were recorded continuously (Swisstom BB2 device, SenTec AG, Landquart, Switzerland) to evaluate silent spaces. ‘Silent spaces’ were defined as the region of interest (ROI) showing impedance changes were less than 10% of maximal impedance changes during tidal ventilation [14, 15]. The amount of silent spaces was expressed as a percentage of the entire lung. The dependent silent spaces are poorly ventilated areas located in dependent lung regions, potentially representing lung collapse and the nondependent silent spaces are poorly ventilated areas located in nondependent lung regions, potentially representing lung overinflation [14, 15]. ‘Increase’ in non-dependent silent spaces when applying high PEEP was calculated as [non-dependent silent spaces at PEEP 15 cmH_2_O] − [non-dependent silent spaces at PEEP 5 cmH_2_O]. ‘Decrease’ in dependent silent spaces when applying high PEEP was calculated as [dependent silent spaces at PEEP 5 cmH_2_O] − [dependent silent spaces at PEEP 15 cmH_2_O].

### Recruitablity

Recruitment-to-inflation ratio (*R*/*I* ratio) was calculated with expiratory tidal volume measured at the time of releasing PEEP 15 to 5 cmH_2_O (or airway opening pressure, either of which was higher) in both positions. Patients were divided into high recruiter or low recruiter. High recruiter was defined as patients with *R*/*I* ratio over the median value measured in supine position.

### Statistical analysis

Data are expressed as median and interquartile range (IQR). Mann–Whitney *U* tests (or Fisher’s exact tests for categorical data) were used to compare the differences between high recruiter versus low recruiter. *R*/*I* ratio measured in supine position and in prone position were compared using paired-*t* test. One-way analysis of variance for repeated measures followed by Tukey’s multiple comparison test was used to evaluate the effect of each condition on variables. Sensitivity analysis was conducted to investigate if the selected cut-off value of 0.68 was robust to separate patients into high versus low recruiter in each parameter. The Pearson’s correlation was used to test the relationship between *R*/*I* ratio and changes in respiratory parameters when increasing PEEP in supine and prone position. All tests were 2-tailed, and differences were considered significant when *p* ≤ 0.05.

## Results

### Baseline patient demographics

#### Overall

A total of 43 patients with COVID-19-associated ARDS was consecutively included. Additional measurements of EIT were performed in four patients who required reintubation after the first measurements. Three measurements were excluded due to malfunction of EIT from 47 measurements and thus data from 44 measurements were analyzed (Additional file 2: Figure S1). Baseline patient demographics, respiratory parameters, and the analysis of CT data on admission are described in Table 1.

**Table 1:** Demographics of patients

	All (*n* = 43)	High recruiter (*n* = 22)	Low recruiter (*n* = 21)	*p* value*
Age, years	73 (62–78)	72 (61–79)	73 (72–76)	0.70
Sex, *M*, *n* (%)	29 (67)	11 (50)	18 (86)	0.01
Height, cm	168 (160–170)	164 (156–170)	170 (165–172)	0.05
BMI, kg/m^2^	23 (22–26)	23 (22–27)	23 (22–25)	0.68
APACHE2 score	16 (14–20)	16 (14–19)	17 (14–20)	0.93
MV days before ICU admission, days	0 (0–0)	0 (0–0)	0 (0–0)	0.94
Severity of ARDS, *n* (%)				0.12
Mild	10 (23)	3 (14)	7 (33)	
Moderate	27 (63)	14 (64)	13 (62)	
Severe	6 (14)	5 (23)	1 (5)	
Tidal volume, ml/kg	6.0 (5.9–6.0)	6.0 (5.9–6.0)	6.0 (5.9–6.1)	0.87
Total PEEP, cmH_2_O	5.6 (5.4–6.2)	5.6 (5.4–6.1)	5.7 (5.4–6.2)	0.81
Airway opening pressure, *n* (%)	27 (63)	13 (59)	14 (64)	0.33
Airway opening pressure, cmH_2_O^†^	5.9 (5.0–6.3)	6.0 (5.0–6.3)	5.9 (5.1–6.4)	0.85
Respiratory rate, breaths/min	18 (15–20)	20 (18–20)	15 (15–20)	< 0.01
Plateau pressure, cmH_2_O	14 (13–16)	16 (14–17)	14 (13–15)	0.03
Driving pressure, cmH_2_O	9 (8–10)	10 (8–11)	8 (7–9)	0.01
Respiratory system compliance, ml/cmH_2_O	43 (33–49)	36 (29–44)	47 (43–56)	< 0.01
Recruitment to Inflation ratio in supine	0.68 (0.52–0.84)	0.84 (0.78–0.99)	0.51 (0.39–0.58)	< 0.01
Recruitment to Inflation ratio in prone	0.69 (0.54–0.86)	0.83 (0.72–0.94)	0.53 (0.37–0.65)	< 0.01
PaO_2_/FiO_2_ at Baseline, mmHg	174 (135–191)	170 (111–189)	179 (153–226)	0.19
pH	7.33 (7.27–7.37)	7.32 (7.25–7.35)	7.34 (7.29–7.40)	0.16
PaCO_2_, mmHg	50 (42–54)	52 (45–55)	47 (41–53)	0.26
Dead space ventilation, %	17 (9–24)	18 (14–27)	16 (9–21)	0.30
CT analysis, %				
Hyper inflated	2 (1–8)	2 (1–3)	7 (1–12)	0.06
Normally aerated	63 (58–72)	60 (54–65)	69 (60–73)	0.05
Poorly aerated	15 (13–21)	21 (16–24)	14 (10–15)	< 0.01
Non aerated	12 (9–17)	14 (10–19)	11 (7–14)	0.03
ICU Mortality,* n* (%)	4 (9)	2 (9)	2 (10)	1.00
Length of ICU stay, days	14 (8–39)	21 (14–39)	9 (7–21)	0.04

Data are presented as median (interquartile range) unless otherwise indicated

BMI, body mass index; APACHE2, Acute Physiology, Age and Chronic Health Evaluation; MV, mechanical ventilation; ARDS, acute respiratory distress syndrome; PEEP, positive end-expiratory pressure; PaO_2_, partial pressure of arterial oxygen; FiO_2_, fraction of inspired oxygen; PaCO_2_, partial pressure of carbon dioxide

^*^p values refer to the comparison between the high and low recruiters

^†^Airway opening pressure was measured in supine position

P_a_O_2_/F_I_O_2_ was 174 mmHg (interquartile range [IQR] 135–191) and respiratory system compliance was 43 ml/cmH_2_O (IQR 32–49). *R*/*I* ratio measured in supine position was 0.68 (IQR 0.52–0.84) and varied among patients. The sensitivity analysis showed that *R*/*I* ratio between 0.58 and 0.68 produced the statistically same results as using the median value of *R*/*I* ratio when separating high recruiter versus low recruiter, in terms of the impacts of PEEP on oxygenation, respiratory compliance and silent spaces (Additional file 2: Figure S2). Based on the previous studies [16, 17], therefore, patients were classified into two groups by using the median value of *R*/*I* ratio (measured in supine position) found in this cohort to determine lung recruitablity: *high recruiter* (*R*/*I* ${ \geqq }$ 0.68) versus* low recruiter* (*R*/*I* < 0.68).

**Fig. 1:**
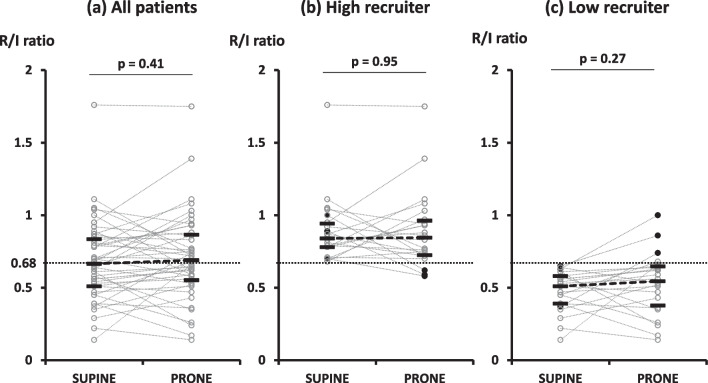
The effects of positioning on individual values of recruitment-to-inflation ratio. Black bars represent median and interquartile range. *R*/*I* ratio was not altered by changing body position in all patients (**a**), high recruiter (**b**), and low recruiter (**c**). Black circles show the patients who changed *R*/*I* ratio across a median value of 0.68 from supine position to prone position. *R*/*I*, ratio recruitment-to-inflation ratio

Overall, *R*/*I* ratio was not altered by changing position in all patients, high, and low recruiter. (Fig. 1). In three patients of each group (low recruiter and high recruiter), *R*/*I* ratio was altered across the median value of 0.68 by changing body position.

#### High recruiter versus low recruiter

*R*/*I* ratios measured in supine position and prone position were significantly higher in high recruiter versus low recruiter, *as per* definition (0.84 [0.78–0.99] vs. 0.51 [0.39–0.58] in supine; *p* < 0.01, 0.83 [0.72–0.94] vs. 0.53 [0.37–0.58] in prone; *p* < 0.01). Oxygenation was the same between high recruiter and low recruiter (170 [111–189] vs. 179 [153–226] mmHg; *p* = 0.19) and the severity of ARDS was also the same, but respiratory system compliance was significantly lower in high recruiter versus low recruiter (36 [29–44] vs. 47 [43–56] ml/cmH_2_O; *p* < 0.01) and thus both plateau pressure and driving pressure were significantly higher in high recruiter versus low recruiter. The affected lung regions, i.e*.*, the amount of poor aeration and non-aeration in CT was significantly larger in high recruiter versus low recruiter (poor aeration 21 [16–24] vs. 14 [10–15]%; *p* < 0.01, non-aeration 14 [10–19] vs. 11 [7–14]%; *p* = 0.03, respectively). The length of ICU stay was significantly longer in high recruiter versus low recruiter (Table 1).

### Prediction of response to PEEP using *R*/*I* ratio

**Fig. 2:**
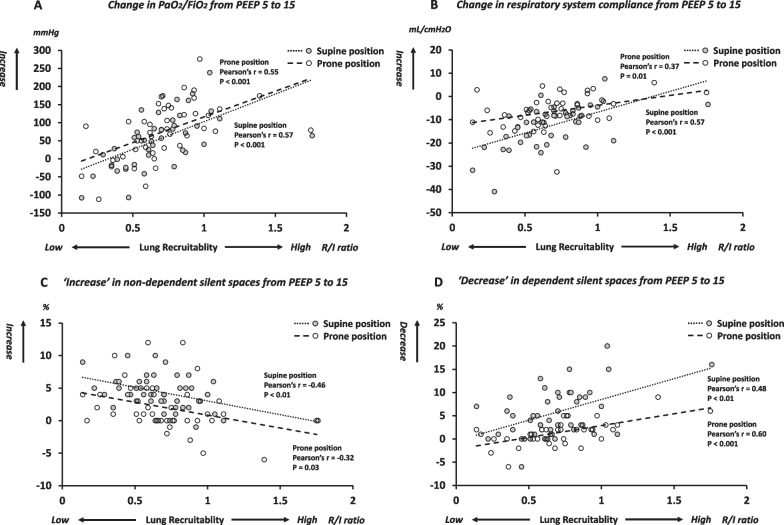
Correlation between *R*/*I* ratio and **A** ‘increase’ in PaO2/FiO2, **B** ‘increase’ in respiratory system compliance, **C** ‘increase’ in non-dependent silent spaces, **D** ‘decrease’ in dependent silent spaces when applying high PEEP in each body position. *R*/*I* ratio was measured when releasing PEEP from 15 to 5 cmH_2_O in each position. Grey-colored circle and white-colored circle represent values obtained from supine position and prone position, respectively. The black short-dot line and long-dot line represent the linear regression in supine position and in prone position. In both positions, the higher *R*/*I* ratio was, the more PaO2/FiO2 improved (**A**), the more respiratory system compliance improved (**B**), the less non-dependent silent spaces increased (**C**), and the more dependent silent spaces decreased (**D**), when applying high PEEP. PEEP, positive end expiratory pressure; *R*/*I*, ratio recruitment-to-inflation ratio

*R*/*I* ratio was positively correlated with the improvement in PaO_2_/FiO_2_ when applying high PEEP, both in supine and prone position (Pearson’s *r* = 0.57, 0.55; *p* < 0.001, *p* < 0.001, respectively: Fig. 2A). *R*/*I* ratio was also positively correlated with the improvement in respiratory system compliance when applying high PEEP, both in supine and prone position (Pearson’s *r* = 0.57, 0.37; *p* < 0.001, *p* = 0.01, respectively: Fig. 2B).

*R*/*I* ratio was inversely correlated with the increase in non-dependent silent spaces when applying high PEEP, both in supine and prone position (Pearson’s *r* = − 0.46, − 0.32; *p* < 0.01, *p* = 0.03, respectively: Fig. 2C); was positively correlated with the decrease in dependent silent spaces when applying high PEEP, both in supine and prone position (Pearson’s *r* = 0.48, 0.60; *p* < 0.01, *p* < 0.001, respectively: Fig. 2D).

### Impacts of PEEP and position

#### High recruiter

**Fig. 3:**
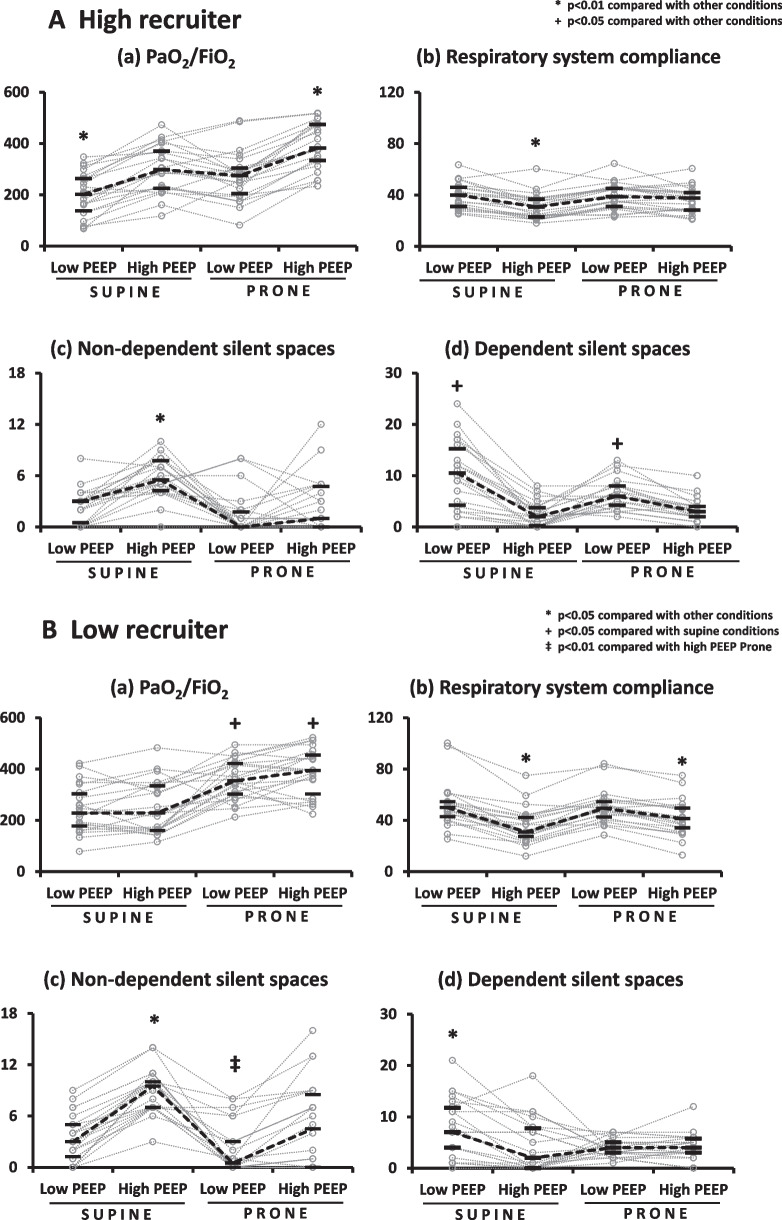
The impacts of body position and PEEP on gas exchange, respiratory mechanics and silents spaces in EIT in **A** high recruiter and **B** low recruiter. Black bars represent median and interquartile range. **A**-high recruiter Oxygenation was lowest in low PEEP + supine and highest in high PEEP + prone (**a**). Respiratory system compliance was worst in high PEEP + supine (**b**). This is because high PEEP in supine position caused the largest amount of non-dependent silent spaces in EIT (presumably lung overinflation) (**c**). The amount of dependent silent spaces in EIT (presumably lung collapse) was largest in low PEEP + supine and least in high PEEP conditions (**d**). *p < 0.01 compared with other conditions, +*p* < 0.05 compared with other conditions. **B**-low recruiter High PEEP (vs. low PEEP) did not improve oxygenation both in supine position and prone position. Prone position per se achieved the highest oxygenation, independent of PEEP levels (**a**). High PEEP (vs. low PEEP) worsened respiratory system compliance in supine position and prone position (**b**), because high PEEP increased the amount of non-dependent silent spaces in EIT (presumably lung overinflation) in supine position and prone position (**c**). The amount of dependent silent spaces in EIT (presumably lung collapse) was highest in low PEEP + supine and it was similar among rest of conditions (**d**). *p < 0.01 compared with other conditions, + *p* < 0.01 compared with supine conditions, ‡*p* < 0.01 compared with high PEEP + prone. EIT, electrical impedance tomography; PEEP, positive end expiratory pressure

Tidal volume was low and similar (volume-controlled ventilation ≈ 6.0 mL/kg) in all groups (Additional file 1: Table S1). In supine position, high PEEP (vs. low PEEP) improved oxygenation (298 [226–370] vs. 202 [138–263] mmHg, *p* < 0.01: Fig. 3A-a) and decreased the amount of dependent (dorsal) silent spaces (presumably lung collapse: 2 [0–4]%, *p* < 0.05 vs. low PEEP groups: Fig. 3A-d), but simultaneously achieved the highest amount of non-dependent (ventral) silent spaces (presumably lung overinflation: 6 [4–8]%, *p* < 0.01 vs. all: Fig. 3A-c) and thus resulted in worst respiratory system compliance (31 [23–37] ml/cmH_2_O, *p* < 0.01 vs. all: Fig. 3A-b), the highest driving pressure (Additional file 1: Table S1). PaCO_2_ was higher and pH was lower in high PEEP + supine (vs. low PEEP conditions).

**Fig. 4:**
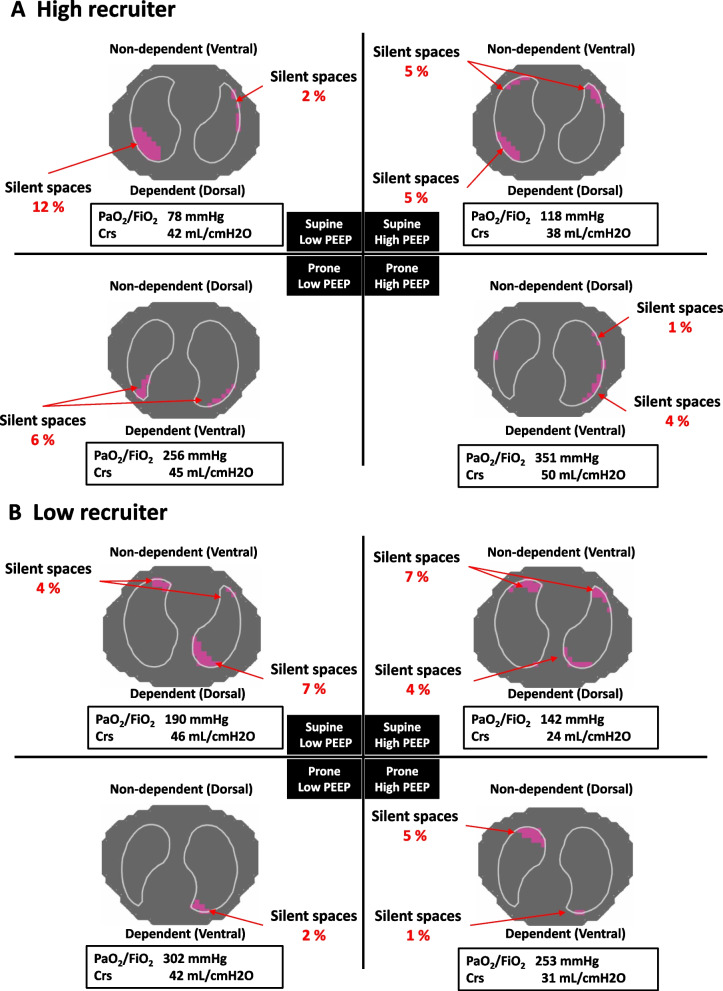
Representative EIT images in **A** high recruiter and **B** low recruiter. Representative EIT images showing the amount of silent spaces, i.e*.*, poorly ventilated areas, in conjunction with gas exchange and respiratory system compliance are presented in all conditions. EIT, electrical impedance tomography; PEEP, positive end expiratory pressure; Crs, respiratory system compliance

Prone position per se improved oxygenation (prone vs. supine at low PEEP: 275 [204–304] vs. 202 [138–263] mmHg, *p* < 0.01: Fig. 3A-a) and decreased the amount of dependent silent spaces without increasing the amount of non-dependent silent spaces. A combination of prone position with high PEEP achieved the highest oxygenation (383 [334–474] mmHg, *p* < 0.01 vs. all groups: Fig. 3A-a) without worsening respiratory system compliance (Fig. 3A-b). Prone position with high PEEP decreased the amount of dependent (ventral) silent spaces (3 [2–4]%; *p* < 0.05 vs. low PEEP groups: Fig. 3A-d) without increasing the amount of non-dependent (dorsal) silent spaces (Fig. 3A-c). A representative case of high recruiter is presented in Fig. 4A.

#### Low recruiter

Tidal volume was low and similar (volume-controlled ventilation ≈ 6.0 mL/kg) in all groups (Additional file 1: Table S1). Both in supine position and in prone position, high PEEP (vs. low PEEP) did not improve oxygenation (high PEEP vs. low PEEP in supine: 229 [160–334] vs. 228 [178–303] mmHg, *p* = 0.98; high PEEP vs. low PEEP in prone: 395 [303–454] vs. 354 [302–422] mmHg, *p* = 0.36: Fig. 3B-a), but increased the amount of non-dependent silent spaces (high PEEP vs. low PEEP in supine: 10 [7–10] vs. 3 [1–5]%, *p* < 0.01; high PEEP vs. low PEEP in prone: 5 [0–9] vs. 1 [0–3]%, *p* < 0.01: Fig. 3B-c), worsened respiratory system compliance (high PEEP vs. low PEEP in supine: 31 [27–42] vs. 50 [43–54] ml/cmH_2_O, *p* < 0.05; high PEEP vs. low PEEP in prone: 41 [34–50] vs. 49 [43–54] ml/cmH_2_O in prone, *p* < 0.05: Fig. 3B-b) and increased driving pressure (Additional file 1: Table S1). PaCO_2_ was higher and pH was lower both in high PEEP + supine and in high PEEP + prone (vs. low PEEP conditions).

Instead of increasing PEEP, changing position from supine to prone (i.e*.*, low PEEP + prone) achieved better oxygenation (vs. supine groups, *p* < 0.01: Fig. 3B-a), less dependent silent spaces (vs. low PEEP + supine, *p* < 0.05: Fig. 3B-d), and less non-dependent silent spaces (vs. high PEEP + supine, *p* < 0.05; vs. high PEEP + prone, *p* < 0.01: Fig. 3B-c). A representative case of low recruiter is presented in Fig. 4B.

## Discussion

Our study confirmed that lung recruitablity (measured by a simple bedside technique, *R*/*I* ratio) varies in patients with COVID-19-associated ARDS. Also, *R*/*I* ratio may be useful to personalize ventilatory strategies in terms of PEEP in COVID-19-associated ARDS. *First*, high PEEP in prone position achieved the highest oxygenation and decreased the amount of dependent silent spaces, i.e*.*, presumably lung collapse (vs. low PEEP in both positions) without increasing the amount of non-dependent silent spaces, i.e*.*, presumably overinflation in patients with high lung recruitablity. *Second*, low PEEP in prone position achieved better oxygenation (vs. both PEEPs in supine position), less amount of dependent silent spaces (vs. low PEEP in supine position), and less amount of non-dependent silent spaces (vs. high PEEP in both positions) in patients with low lung recruitablity.

### Heterogeneity of COVID-19-associated ARDS

Since the heterogeneity has been recognized as an inherent nature of ARDS, effort have been made to identify subgroups of patients from biological, physiological, or morphological perspective and to propose the personalized strategy on the basis of identified phenotypes [3, 18]. The same attempt has been made in patients with COVID-19-associated ARDS, e.g*.*, type L versus type H [5, 19, 20]; but so far none of studies has successfully shown to identify distinct phenotypes and thus proposed the personalized strategy from the physiological point of view [21].

Recently, *R*/*I* ratio has been developed as a simple bedside technique to identify the patients who has the potential for lung recruitment [4]. In the current study, the value of *R*/*I* ratio varied widely from 0.14 to 1.76 (range), suggesting that the potential for lung recruitment was largely different patient by patient in COVID-19-associated ARDS. Of note, lung recruitablity could not be predicted by the initial oxygenation on ICU admission nor the severity of ARDS *as per* Berlin definition in our study (Table 1). Indeed, a previous study using EIT reported that PEEP settings according to traditional oxygenation criteria, i.e*.*, PEEP/F_I_O_2_ table were not adequate but were either insufficient to cause lung collapse or excessive to cause hyperinflation in patients with COVID-19-associated ARDS [9]. Therefore, our attempt to identify the patients who may or may not benefit from lung recruitment, i.e*.*, higher PEEP or prone position or both, by means of a simple bedside technique, *R*/*I* ratio, may be useful to maximize lung protection and minimize VILI in ARDS.

### Impact of PEEP and position: high recruiter

The current study revealed that a combination of high PEEP with prone position decreased dependent silent spaces (presumably lung collapse) without increasing non-dependent silent spaces (presumably lung overinflation), resulting in better oxygenation and better respiratory mechanics in patients with high lung recruitablity. This is because prone position can maximize the benefits of high PEEP on recruiting lungs; and minimize the adverse effects of high PEEP on cardiopulmonary system. We found that even in high recruiter, high PEEP in supine position had risks of increasing overinflation, worsening respiratory system compliance, and deteriorating hemodynamics. Such adverse effects of high PEEP on cardiopulmonary system were canceled by changing position from supine to prone. Several plausible explanations are offered.

*First*, a previous experimental study confirmed that compared with supine position, prone position decreased pleural pressure gradient by increasing cephalo-caudal dimension and decreasing antero-posterior dimension of the lungs, achieving more homogeneous distribution of ventilation, and decreasing hyperinflation [22]. *Second*, previous studies reported that prone position increased arterial pressure, cardiac output and decreased heart rate [23, 24]. This is explained at least in a part by normalization of right ventricle function [25]. Thus, in patients with high recruitablity, prone position can maximize the benefits, minimize the adverse effects of high PEEP.

### Impact of PEEP and position: low recruiter

Low recruiter was more likely to suffer from the adverse effects of high PEEP than high recruiter. When applying high PEEP, the amount of non-dependent silent spaces was greater in low recruiter than in high recruiter, regardless of body position (× *2* in supine position: Fig. 3B-c vs. Fig. 3A-c, × *4* in prone position: Fig. 3B-c vs. Fig. 3A-c). Our EIT findings were corroborated by the facts that in low recruiter, high PEEP (vs. low PEEP) did not improve oxygenation but worsened respiratory system compliance in both body positions. Thus applying high PEEP in the absence of lung recruitablity seems to cause harm rather than benefit and overall, prone position did not improve lung recruitablity (Fig. 1) nor cancel the adverse effects of high PEEP in low recruiter (Fig. 3B). This is in accordance with previous CT findings showing that higher PEEP caused more harm by increasing the amount of hyperinflated lung tissue and alveolar strain in patients with low recruitablity (vs. high recruitablity) [26, 27]. Thus, higher PEEP should be avoided in low recruiter, independent of body position.

Our study found that in low recruiter, a combination of prone position with low PEEP (but not high PEEP) was effective to maintain less non-dependent silent spaces and less dependent silent spaces (Fig. 3B-c, d). However, the impacts of prone position in low recruiter seem to be inconsistent. It is reported that the benefits of prone position were confined to high recruiter in small studies with COVID-19-associated ARDS [16]. Different numbers of patients enrolled in the study or different time points of measurements in each study may explain inconsistent findings. Since protective mechanisms of prone position by homogenizing pleural pressure gradient and lung stress were proven to occur universally, not only in injured lungs but in normal lungs [22, 28, 29], it is reasonable to think that the physiological benefits of prone position would be observed (to different degree) irrespective of lung recruitablity and severity of lung injury. Currently prone position is recommended in severe ARDS, but of note, the indication of prone position has been expanding and it has been widely used in various degree of lung injury in COVID-19-associated ARDS, e.g*.*, ≈ 60% of mild ARDS, awake prone position before intubation [30, 31].

### Utility of R/I ratio to predict response to PEEP

*R*/*I* ratio calculated by expired lung volume in a ventilator has at least two potential concerns: (1) inaccurate volume measurement due to gas leakage and/or performance of flow measurement inherent in each ventilator; (2) no validation of *R*/*I* ratio to predict responses to PEEP with lung imaging techniques. The utility of *R*/*I* ratio to predict response to PEEP was confirmed with various parameters, i.e*.*, oxygenation, respiratory system compliance, and lung imaging technique of EIT. Our data suggest that *R*/*I* ratio can reasonably predict responses to PEEP (Fig. 2): the higher *R*/*I* ratio was, the less overinflation was induced, the more collapsed lungs were recruited, and the more oxygenation was improved when applying high PEEP. It is also important to stress that responses to PEEP was not altered by changing position (Fig. 1).

Therefore, our data provide strong evidence for potential utility of *R*/*I* ratio in COVID-19-associated ARDS. *R*/*I* ratio can be a reasonable index at the bedside to predict the efficacy (recruiting lungs) and risk (increasing overinflation) of ‘body position’ and ‘PEEP’. *R*/*I* ratio may be useful to personalize ventilatory strategy in terms of PEEP in COVID-19-associated ARDS.

### Limitation

This study has several limitations. *First*, it is a small sample size enrolled from a single center. In addition, our goal is short-term, and a prospective study is necessary to evaluate the impact of our proposed strategy on long-term outcome. This is cross-over design and each of four sequences were not randomized, which might not exclude completely the carry-over effects with at least 30-min washout period. Of course, a prospective study is needed to evaluate the plausibility of this personalized ventilatory strategy in COVID-19-associated ARDS. *Second*, the utility of *R*/*I* ratio was carefully validated with different techniques. Silent spaces reflecting poorly ventilated areas in EIT is interpreted as overinflation if appeared in non-dependent lung regions and lung collapse if appeared in dependent lung regions, as previously described [14, 15]. In volume-controlled ventilation, non-dependent silent spaces might appear by improving dependent lung compliance without any change in local compliance of non-dependent lung regions (i.e*.*, the redistribution of ventilation from non-dependent lung to dependent lung). But lower respiratory system compliance was accompanied by larger amount of non-dependent silent spaces (Fig. 3), precluding such concern. Corresponding morphological data with CT are lacking in this study. *Third*, majority of patients were moderate ARDS *as per* the Berlin definition [12] and non-obese in our study cohort. This might partially explain that median *R*/*I* ratio was different among each study [16]. Thus, caution is necessary to extrapolate our findings into different clinical contexts, e.g*.*, more severe ARDS, obese patients.

## Conclusion

Lung recruitablity varies in patients with COVID-19-associated ARDS. *R*/*I* ratio may be useful to personalize PEEP in COVID-19-associated ARDS. Higher PEEP in prone position and lower PEEP in prone position decreased the amount of dependent silent spaces (suggesting lung collapse) without increasing the amount of non-dependent silent spaces (suggesting overinflation) in high recruiter and in low recruiter, respectively.

## Supplementary Information

**Additional file 1.** Supplemental Methodology and Table.

**Additional file 2.** Supplemental Figures.

## Data Availability

The datasets used and/or analyzed during the current study are available from the corresponding author on reasonable request.
