# Supplementary material for: Personalized ventilatory strategy based on lung recruitablity in COVID-19-associated acute respiratory distress syndrome: a prospective clinical study
Source: Crit Care. 2023 Apr 19;27:152. doi: 10.1186/s13054-023-04360-6 (PMC10116825; doi:10.1186/s13054-023-04360-6)
Supplement: Supplementary file 1 — Additional file 1. Supplemental Methodology and Table. [file 13054_2023_4360_MOESM1_ESM.docx]

**SUPPLEMENTARY Appendix**

**List of contents**

**APPENDIX 1- Methodology**………………………………………………………...2

**APPENDIX 2- Supplemental table**……………………………………………….....6

**APPENDIX 3- Supplemental figure legends**………………………………………..7

**REFERENCES**…………………………………………..……………………..……..8

**Apendix-1**

**METHODS**

***Study population:*** This prospective observational study was carried out in a single thirty-bed intensive care unit (ICU) at Osaka University Hospital following the approval from the Ethics committee for Clinical Studies, Osaka University Hospital, Suita, Japan (No.20039). The study has been registered on ClinicalTrial.gov (ID NCT04473300). Forty-three consecutive patients were then enrolled in between May 2020 and February 2021. The written informed consent from each patient or substitute decision-maker was waived for this study. Patients were eligible if SARS-CoV-2 infection was positive, defined as being positive in real time reverse transcriptase-polymerase chain reaction assay using nasal or pharyngeal swab samples; age was ≧18 years old; met criteria for ARDS *as per* the Berlin definition(1). Patients reintubated after first enrollment were also included for additional measurements of EIT (***Figure 1***). Exclusion criteria were contraindication for EIT monitoring (*e.g.*, unstable spine or pelvic fractures; pacemaker, automatic implantable cardioverter defibrillator; and skin lesions between the 4^th^ and 5^th^ ribs where the EIT belt is worn); home mechanical ventilation before inclusion; pregnancy; do-not-resuscitate; and increased intracranial pressure. Demographics were obtained from the patient’s medical file on ICU admission, *e.g.*, age, gender, height, weight, admission diagnosis, comorbidities, ICU admission date, intubation date, and clinical severity as assessed by the Acute Physiology and Chronic Health Evaluation II (APACHE II) score.

***CT scans:*** Thorasic CT scans were obtained (SOMATOM Definition Flash, Siemens Medical Solutions, Erlangen, Germany) upon ICU admission in all patients. Unenhanced helical CT scans (matrix: 512 rows×512 columns; slices 5.0 mm; voltage 120 kV; tube current adjusted by automatic exposure control) were performed. The images were analyzed using Aquarius iNtuition (Terarecon, Durham, NC) for segmentation and volumetric measurements. The segmentation with a region-growing algorithm was performed semiautomatically. The heart and diaphragm were excluded from the regions of interest. The volume of hyperinflation, normal aeration, poor aeration, and non-aeration was calculated respectively. Each lung compartment was identified, according to densities expressed in Hounsfield Unit: non–aeration (density between +100 and –200), poor aeration (density between –201 and –500), normal aeration (density between –501 and –900), and hyperinflation (density between –901 and –1000)(2).

***Protocol:*** Prior to initiating the measurements, all patients were deeply sedated with sedatives and/or opioids and paralyzed with intravenous administration and a continuous infusion of rocuronium. The level of sedation was quantitatively evaluated by using Bispectral index (BIS: Aspect Medical Systems, Norwood, USA) to confirm patients were deeply sedated throughout the measurements. All patients were confirmed to have no signs of unstable hemodynamics by clinicians before measurements and carefully evaluated the hemodynamic change (*e.g.,* heart rate, arterial pressure, and central venous pressure) throughout the measurements.

Patients were then sequentially assigned to each of four conditions as follows:

- High PEEP, Supine;
- Low PEEP, Supine;
- High PEEP, Prone;
- Low PEEP, Prone;

All patients were ventilated with assisted volume-controlled mode, targeting tidal volume of 6 ml/kg predicted body weight, respiratory frequency of 35 min^-1^ or less targeted to pH 7.20-7.45, inspiratory time of 0.6-1.2 sec. Airway opening pressure was identified by a pressure-volume curve on the ventilator at low constant flow, as described previously(3). High PEEP and low PEEP was defined as 15 cmH_2_O and 5 cmH_2_O (or airway opening pressure, either of which was higher) respectively. All measurements were conducted within 24 hours after ICU admission and, for the patients who required reintubation, within 24 hours after reintubation. Each measurement (*e.g.*, arterial blood gas analysis, respiratory parameters, hemodynamics, EIT measurements) was performed at least 10 minutes after changing ventilator settings and at least 30 minutes after changing body positions.

***Electric Impedance Tomography:*** EIT data were recorded continuously with 32 electrodes placed around the chest at the level of the fifth intercostal space (Swisstom BB2 device, SenTec AG, Landquart, Switzerland) throughout the protocol to evaluate silent spaces. Reconstructed EIT images represent relative impedance changes for each pixel (delta Z), compared to a convenient reference taken at the beginning of data acquisition(4). ‘Silent spaces’ were defined as the region of interest (ROI) showing impedance changes were less than 10% of maximal impedance changes during tidal ventilation(5, 6). The amount of silent spaces was expressed as a percentage of the entire lung. The image of lung was divided into two equal zones, non-dependent half and dependent half, where each zone comprised 50% of the ventro-dorsal distance, and encompassed the complete area of the lung encircled by the band. The dependent silent spaces are poorly ventilated areas located in dependent lung regions, potentially representing lung collapse and the nondependent silent spaces are poorly ventilated areas located in nondependent lung regions, potentially representing lung overinflation(5, 6). ‘Increase’ in non-dependent silent spaces when increasing PEEP was calculated as [value of non-dependent silent spaces at PEEP 15cmH_2_O] minus [value of non-dependent silent spaces at PEEP 5cmH_2_O]. ‘Decrease’ in dependent silent spaces when increasing PEEP was calculated as [value of dependent silent spaces at PEEP 5cmH_2_O] minus [value of dependent silent spaces at PEEP 15cmH_2_O]. Representative values of silent spaces were used from 3-minute recordings of EIT data.

***Recruitablity:*** Recruitment-to-inflation ratio (R/I ratio) was calculated with expiratory tidal volume measured at the time of releasing PEEP 15 to 5 cmH_2_O (or airway opening pressure, either of which was higher) in both positions. Patients were divided into high recruiter or low recruiter. High recruiter was defined as patients with R/I ratio over the median value measured in supine position.

During data collection, physicians wear personal protective equipment according to World Health Organization guideline (N95 or HALO mask, goggles, face shield, gown, gloves, and cap). The measurements were performed with minimum number of people and measurement time. The ventilator used in the current study was NPB 840 (Puritan Bennett, Pleasanton, CA) or NPB 980 (Puritan Bennett, Pleasanton, CA), either of which was available when the patients were admitted to the ICU. All pressure and volume data were derived from a ventilator display screen.

***Definitions***: Definitions of pulmonary pressures and EIT measurements was as follows

- Plateau pressure, airway pressure measured during 0.5 second of inspiratory hold (*i.e*. zero flow phase)
- Total PEEP, airway pressure measured during a short expiratory hold (*i.e*. zero flow phase)
- Driving Pressure = [Plateau pressure – total PEEP] cmH_2_O
- Compliance of the respiratory system = [tidal volume/(driving Pressure)] mL·cmH_2_O^-1^
- ‘Increase’ in non-dependent silent spaces when applying high PEEP= [non-dependent silent spaces at PEEP 15cmH_2_O] – [non-dependent silent spaces at PEEP 5cmH_2_O].
- ‘Decrease’ in dependent silent spaces when applying high PEEP = [dependent silent spaces at PEEP 5cmH_2_O] – [dependent silent spaces at PEEP 15cmH_2_O].

***Statistical analysis:*** The study was exploratory and sample size was not formally calculated. Data are expressed as median and interquartile range (IQR). Mann-Whitney U tests (or Fisher’s exact tests for categorical data) were used to compare the differences between high recruiter *vs*. low recruiter. R/I ratio measured in supine position and in prone position were compared using paired-t test. 1-way analysis of variance (ANOVA) for repeated measures was used to evaluate the effect of each condition on variables. In the *post hoc* analysis, a Tukey’s multiple comparison test was used to determine condition differences. Sensitivity analysis was conducted to investigate if the selected cut-off value of 0.68 was robust to separate patients into high *vs.* low recruiter in each parameter. The Pearson’s correlation was used to test the relationship between R/I ratio and changes in respiratory parameters when increasing PEEP in supine and prone position. All tests were 2-tailed, and differences were considered significant when p ≤ 0.05.

**Apendix-3 SUPPLEMENTAL TABLE**

**s-Table 1 Respiratory parameters in all conditions: High recruiter and low recruiter**

|  | High recruiter | | | | Low recruiter | | | |
| --- | --- | --- | --- | --- | --- | --- | --- | --- |
|  | Supine | | Prone | | Supine | | Prone | |
|  | Low PEEP | High PEEP | Low PEEP | High PEEP | Low PEEP | High PEEP | Low PEEP | High PEEP |
| Tidal volume, ml/kg | 6.0 (5.9-6.1) | 6.0 (5.9-6.0) | 6.0 (5.9-6.1) | 6.0 (5.9-6.0) | 6.0 (5.9-6.1) | 6.0 (5.9-6.0) | 6.0 (5.9-6.1) | 6.0 (5.9-6.1) |
| Total PEEP, cmH_2_O | 5.8 (5.5-6.0) | 16 (15-16)^†^ | 5.7 (5.4-6.1) | 16 (15-16)^†^ | 5.7 (5.4-6.0) | 15 (15-16)^†^ | 5.7 (5.6-6.1) | 16 (15-16)^†^ |
| Respiratory rate, breaths/min | 22 (19-25) | 22 (19-25) | 22 (19-25) | 22 (20-25) | 19 (16-20) | 19 (16-20) | 19 (17-20) | 19 (17-20) |
| Plateau pressure, cmH_2_O | 15 (14-16) | 27 (26-28)* | 14 (14-16) | 26 (24-27) * | 14 (12-15) | 27 (23-30)^†^ | 14 (13-15) | 26 (23-26)^†^ |
| Driving pressure, cmH_2_O | 9 (8-10) | 11 (10-12)* | 9 (8-10) | 10 (8-11) | 8 (7-9) | 11 (8-14)* | 8 (7-9) | 10 (8-11)* |
| pH | 7.34 (7.29-7.37) | 7.32 (7.28-7.35)^†^ | 7.34 (7.30-7.37) | 7.33 (7.29-7.35) | 7.34 (7.30-7.35) | 7.32 (7.30-7.35)^†^ | 7.34 (7.32-7.38) | 7.32 (7.30-7.36)^†^ |
| PaCO_2_, mmHg | 46 (44-52) | 50 (45-54)^†^ | 47 (43-51) | 48 (45-53) | 47 (44-51) | 49 (45-54)^†^ | 47 (42-50) | 48 (44-52)^†^ |

Data are presented as median (interquartile range).

***Abbreviations***: PEEP positive end-expiratory pressure. PaCO2 partial pressure of carbon dioxide.

*p < 0.05 compared with all,^†^p < 0.05 compared with different PEEP conditions.

**SUPPLEMENTAL FIGURE LEGEND**

**Supplemental Figure 1: Flow chart of patients.**

***Abbreviations***: ARDS acute respiratory distress syndrome; EIT: electrical impedance tomography.

**Supplemental Figure 2: Sensitivity analysis.**

Grey bars represent the range producing the statistically same results as using a median value of R/I ratio (0.68) when separating high *vs.* low recruiter, in terms of the impacts of PEEP on oxygenation, respiratory compliance and silent spaces in supine position and prone position.

***Abbreviations***: Crs respiratory system compliance; DSS dependent silent spaces; NSS nondependent silent spaces.

**REFERENCES**

1. Ranieri VM, Rubenfeld GD, Thompson BT, Ferguson ND, Caldwell E, Fan E, Camporota L, Slutsky AS. Acute respiratory distress syndrome: the Berlin Definition. *Jama* 2012; 307: 2526-2533.

2. Reske AW, Costa EL, Reske AP, Rau A, Borges JB, Beraldo MA, Gottschaldt U, Seiwerts M, Schreiter D, Petroff D, Kaisers UX, Wrigge H, Amato MB. Bedside estimation of nonaerated lung tissue using blood gas analysis. *Critical care medicine* 2013; 41: 732-743.

3. Chen L, Del Sorbo L, Grieco DL, Junhasavasdikul D, Rittayamai N, Soliman I, Sklar MC, Rauseo M, Ferguson ND, Fan E, Richard JM, Brochard L. Potential for Lung Recruitment Estimated by the Recruitment-to-Inflation Ratio in Acute Respiratory Distress Syndrome. A Clinical Trial. *American journal of respiratory and critical care medicine* 2020; 201: 178-187.

4. Victorino JA, Borges JB, Okamoto VN, Matos GF, Tucci MR, Caramez MP, Tanaka H, Sipmann FS, Santos DC, Barbas CS, Carvalho CR, Amato MB. Imbalances in regional lung ventilation: a validation study on electrical impedance tomography. *American journal of respiratory and critical care medicine* 2004; 169: 791-800.

5. Ukere A, März A, Wodack KH, Trepte CJ, Haese A, Waldmann AD, Böhm SH, Reuter DA. Perioperative assessment of regional ventilation during changing body positions and ventilation conditions by electrical impedance tomography. *British journal of anaesthesia* 2016; 117: 228-235.

6. Spadaro S, Mauri T, Böhm SH, Scaramuzzo G, Turrini C, Waldmann AD, Ragazzi R, Pesenti A, Volta CA. Variation of poorly ventilated lung units (silent spaces) measured by electrical impedance tomography to dynamically assess recruitment. *Critical care (London, England)* 2018; 22: 26.

7. Borges JB, Okamoto VN, Matos GF, Caramez MP, Arantes PR, Barros F, Souza CE, Victorino JA, Kacmarek RM, Barbas CS, Carvalho CR, Amato MB. Reversibility of lung collapse and hypoxemia in early acute respiratory distress syndrome. *American journal of respiratory and critical care medicine* 2006; 174: 268-278.
