# Supplementary material for: Personalized ventilatory strategy based on lung recruitablity in COVID-19-associated acute respiratory distress syndrome: a prospective clinical study
Source: Crit Care. 2023 Apr 19;27:152. doi: 10.1186/s13054-023-04360-6 (PMC10116825; doi:10.1186/s13054-023-04360-6)
Supplement: Supplementary file 2 — Additional file 2. Supplemental Figures. [file 13054_2023_4360_MOESM2_ESM.pptx]

## Slide 1
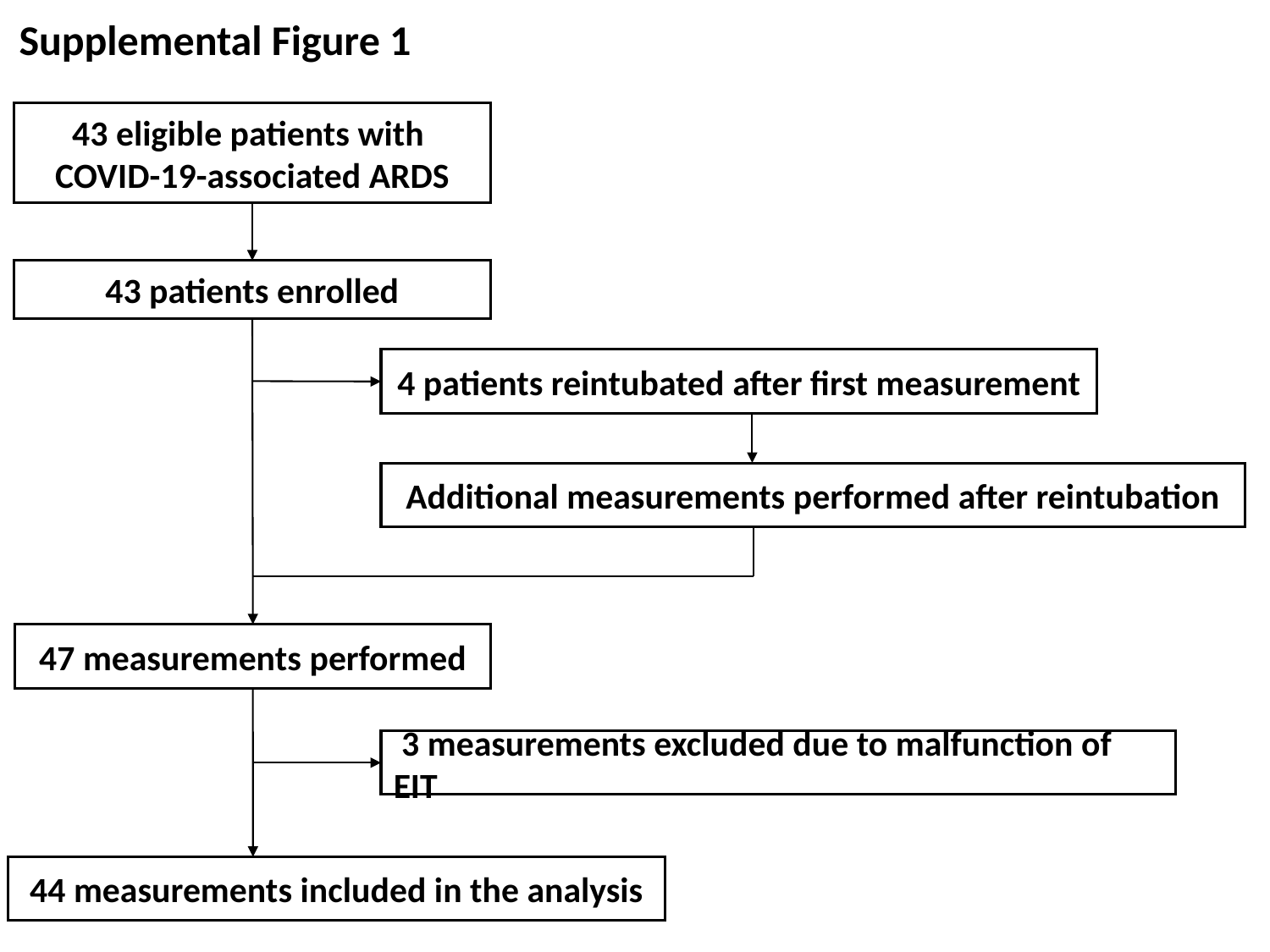

Supplemental Figure 1
43 eligible patients with
COVID-19-associated ARDS
43 patients enrolled
4 patients reintubated after first measurement
Additional measurements performed after reintubation
47 measurements performed
 3 measurements excluded due to malfunction of EIT
44 measurements included in the analysis

## Slide 2
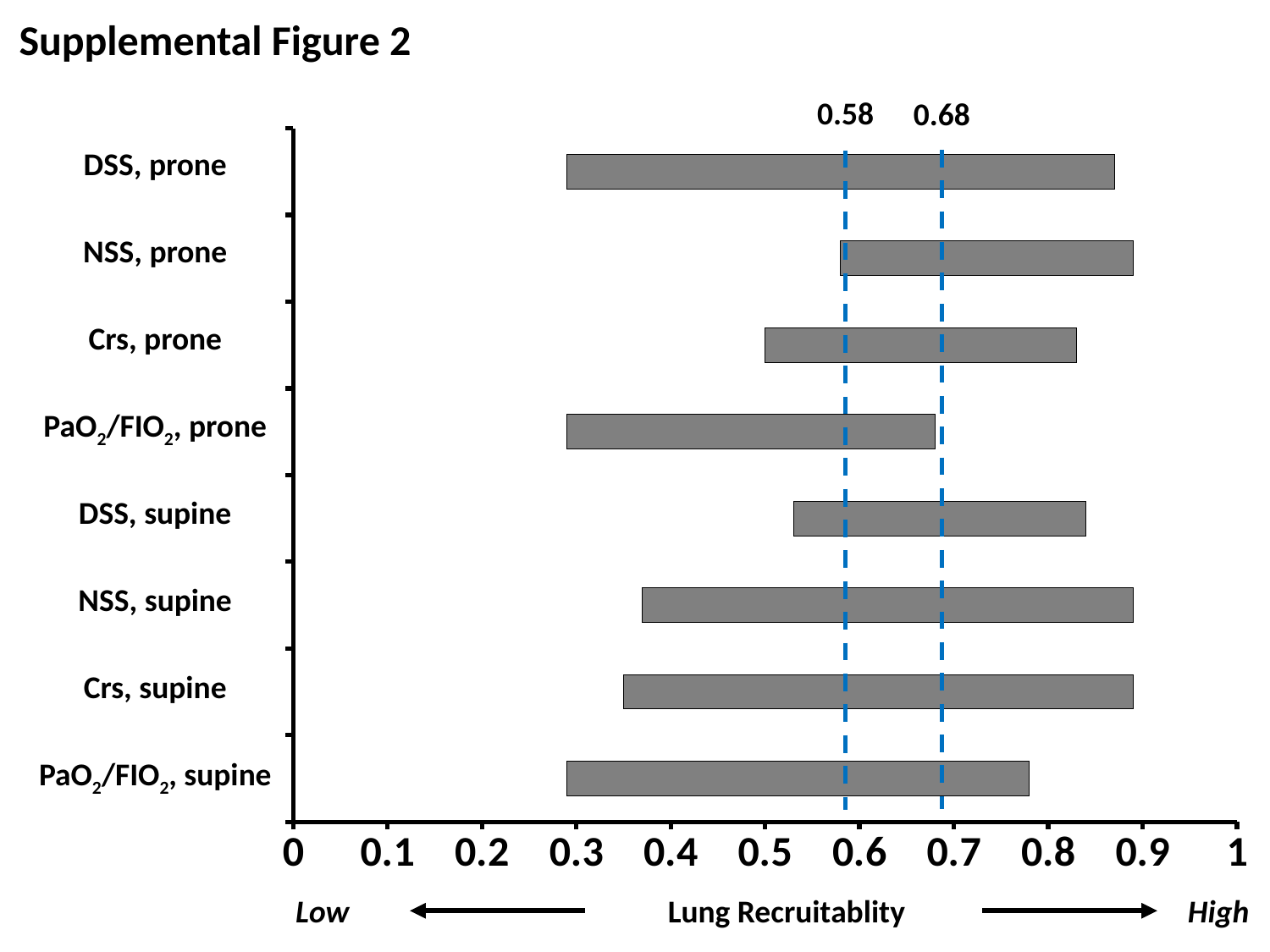

Supplemental Figure 2
0.58
0.68
### Chart
| Category | | | |
|---|---|---|---|DSS, prone
NSS, prone
Crs, prone
PaO2/FIO2, prone
DSS, supine
NSS, supine
Crs, supine
PaO2/FIO2, supine
Low
Lung Recruitablity
High
